# Hepatitis B Virus Sero-Prevalence Among Pregnant Females in Saudi Arabia

**DOI:** 10.4103/1319-3767.39621

**Published:** 2008-04

**Authors:** Mohammed A. Alrowaily, Mostafa A. Abolfotouh, Mazen S. Ferwanah

**Affiliations:** King Fahad National Guard Hospital, King Abdulaziz Medical City, NGHA, Riyadh, Saudi Arabia; 1 Biobanking Section, Research Center, King Abdulaziz Medical City, NGHA, Riyadh, Saudi Arabia

**Keywords:** Antenatal screening, Hepatitis B, postpartum Hepatitis B vaccine

## Abstract

**Background/Aim:**

Since selective screening for Hepatitis B virus (HBV) in pregnant women has failed to identify a high proportion of HBV-infected mothers, pre-natal HBsAg testing of all pregnant women is now recommended. We aimed to determine the prevalence of HBV infection among pregnant women at the ante-natal clinic of a tertiary care center in Saudi Arabia and to identify the target group for postpartum immunization.

**Materials and Methods:**

A total of 755 pregnant females who attended the antenatal clinic from June 2005 to June 2006 for the first time - before 38 weeks of gestation - constituted the target of the present study. Blood samples 30-39 were drawn from all subjects and sera were tested for HBV serologic markers including Hepatitis B surface antigen, anti-HBs, and anti-HBc using ELISA technique (third generation).

**Results:**

The overall prevalence of sero-positive HBsAg among pregnant women was 1.6%. As age increased, the prevalence of sero-positive HBsAg significantly increased (χ^2^ = 116.43, *P* < 0.001), 30–39 were women aged ≥40 were five times more likely to be positive for HBsAg as compared to those <30 years (OR = 4.78). On the other hand, women aged 40 and over were five times more likely to be susceptible to infection with hepatitis as compared to young women aged <20 (OR = 5.15). Women susceptible to HBV infection constituted about 80% of all pregnant females.

**Conclusion:**

These findings reflect that the full impact of the Hepatitis B vaccination program that was conducted in 1989 for all Saudi children has not yet reached all pregnant women, with the majority (79.9%) being nonimmune and thus liable to HBV infection. Postpartum HB immunization should be recommended in such cases.

Hepatitis B virus (HBV) infection is a major public health problem in the Middle East. The majority of the countries in the region have intermediate (2 to <5%) or high (>5%) endemicity of HBV infection.[1] Studies in Saudi Arabia showed prevalence of Hepatitis B surface antigen (HBsAg) that ranges from 7.4 to 17% denoting high endemicity.[2]

The HBV is a major cause of chronic hepatitis, cirrhosis, and hepatocellular carcinoma. It ranks as an important pathogen throughout the world.[3] Transmission occurs mainly from a mother to child at time of parturition, as well as person-to-person (horizontal) transmission among children < 5 years of age.[4] Even when not infected during the perinatal period, children of HBV-infected mothers remain at a high risk of acquiring HBV infection by horizontal transmission during the first 5 years of life.[5]

Since selective screening of pregnant women for HBV has failed to identify a high proportion of HBV-infected mothers,[6] pre-natal HBsAg testing of all pregnant women is now recommended.[7] Universal HBsAg screening of pregnant women to prevent perinatal HBV infection has been shown to be cost saving.[8] Previous studies showed that the prevalence of HBsAg among Saudi pregnant females ranged from 3.9 to 12.7%.[9–11]

A study conducted to investigate the acceptance and efficacy of Hepatitis B immunization in United States women during the postpartum period revealed its feasibility and effectiveness.[12] Thus, the aim of the present study was to determine the prevalence of HBV infection among pregnant women at the ante-natal clinic of a tertiary care center in Saudi Arabia and to identify the target group for postpartum immunization.

## MATERIALS AND METHODS

At the antenatal clinic of King Fahad Hospital at Riyadh National Guard Health Affairs, every pregnant woman was routinely tested in each pregnancy. Serology screening for HBsAg, Hepatitis B core antigen (HBcAb), and Hepatitis B antibody (HBsAb) is requested in every first booking visit. All pregnant females who attended the antenatal clinic from June 2005 to June 2006 for the first time - before 38 weeks of gestation - constituted the subjects of the present study.

Age and parity status were recorded. A total of 755 women were screened for HBsAg, HBsAb, and HBcAb. Blood samples were drawn from all subjects and sera were tested for HBV serologic markers including HBsAg, and HBsAb, and HBcAb were detected by using chemiluminescent microparticle immunoassay using commercial kits from Abbott Laboratories Diagnostics Division, Abbott Park, USA (ABBOTT).

SPSS software program version 10 was used for all statistical analyses.

## RESULTS

Table 1 shows an overall prevalence of sero-positive HBsAg of 1.6%. However, as age increased, the prevalence of sero-positive HBsAg significantly increased (χ^2^ = 116.43, ***P*** < 0.001). Sero-positivity among those aged 40 years and over was seven times as it was among people < 30 (OR = 7.07). Meanwhile, people aged 30 to < 40 are five times more likely to be positive for HBsAg as compared to those < 30 years (OR = 4.78).

**Table 1:** Seroprevalence of HBsAg among pregnant women by age

Age group	HBsAg		OR	Anti-HBs		OR
				
	Negative	Positive		HBs > 10	HBs < 10	
<20 years	25	-		13	12	1.0@
*n* = 25	100%	-		52%	48%	
			1.0@			
20–29 years	212	1		41	172	4.54
*n* = 213	99.9%	0.5%		19.2%	80.8%	
30–39 years	446	9	4.78	87	368	4.58
*n* = 455	98%	2%		19.1%	80.9%	
>40 years	67	2	7.07	12	57	5.15
*n* = 69	97.1%	2.9%		17.4%	82.6%	
Total *n* = 762	750	12		153	609	
	98.4%	1.6%		20.1%	79.9%	
χ^2^	116.43, P< 0.001			59.34, P < 0.001		

@ - Reference category, OR - Odds ratio.

On the other hand, the number of sero-negative cases to Hepatitis B was significantly higher among the elderly compared with the youth. It increased from 48% among those below 20 years to 82.6% among those aged 40 years and more (χ^2^ = 59.34, ***P*** < 0.001). People 30–39 were women aged ≥40 were five times more likely to be susceptible to infection with hepatitis as compared to young people aged < 20 (OR = 5.15). Moreover, those aged 20–40 are about 4.5 times more vulnerable to hepatitis as compared to their counterparts aged < 20 (OR = 4.5).

## DISCUSSION

The present study shows that the sero-prevalence of HBsAg was 1.6%, which is comparable to the <2.6% reported by Al-Mazrou and colleagues[13] and far less than that reported by Al-Shamahy in the study conducted in Yemen[14] and by El-Hazmi.[15] It also shows that 20.1% had HBsAb titer >10 (immunity). This finding was significantly higher among mothers below 20 years of age (52%) who perhaps were exposed to the mass HBV vaccination that took place in 1989 for all Saudi children.[16]

The study also shows that 79.9% of the pregnant females have a nonimmune status making them liable to HBV infection. This suggests that the full impact of the Hepatitis B vaccination program has not yet reached all women during their maternity period, as also reported by a previous study.[13]

Jurema ***et al.***[12] have previously shown that Hepatitis B immunization in the postpartum period is feasible and effective. The availability of a safe and effective hepatitis vaccine[16] encourages us to accelerate viral elimination, and additional intervention such as Hepatitis B immunization in postpartum women can thereon be undertaken. Thus, Hepatitis B immunization can be recommended, giving the first dose immediately on the first postpartum day before the mother gets discharged from the hospital; and the second dose to coincide with her child's first vaccination dose at the age of 2 months, and a third dose to be given to the mother when her child gets vaccinated at the age 6 months. However, further studies to assess the feasibility as well as effectiveness of such a program are necessary.
